# Pintando a História da Cardiologia do Brasil

**DOI:** 10.36660/abc.20201133

**Published:** 2020-12-01

**Authors:** Marcelo Antônio Cartaxo Queiroga Lopes, Gláucia Maria Moraes de Oliveira, Evandro Tinoco Mesquita, Marcelo Dantas Tavares de Melo, Fernando Bacal

**Affiliations:** 1 Hospital Alberto Urquiza Wanderley CabedeloPB Brasil Hospital Alberto Urquiza Wanderley, Cabedelo, PB - Brasil; 2 Universidade Federal do Rio de Janeiro Rio de JaneiroRJ Brasil Universidade Federal do Rio de Janeiro, Rio de Janeiro, RJ – Brasil; 3 Universidade Federal Fluminense NiteróiRJ Brasil Universidade Federal Fluminense, Niterói, RJ - Brasil; 4 Universidade Federal da Paraíba João PessoaPB Brasil Universidade Federal da Paraíba, João Pessoa, PB - Brasil; 5 Universidade de São Paulo Faculdade de Medicina Hospital das Clínicas São PauloSP Brasil Universidade de São Paulo Faculdade de Medicina Hospital das Clínicas Instituto do Coração, São Paulo, SP – Brasil

**Keywords:** Cardiologia/história, Cardiologia/tendências, Cardiologistas, Medicina nas Artes/história, Pinturas

**Figura 1:**
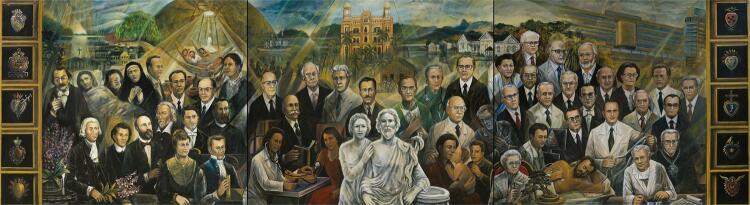
– Pintando a História da Cardiologia do Brasil.

O mural que o artista plástico Flávio Tavares criou para a Sociedade Brasileira de Cardiologia (SBC), O Coração dos Trópicos, é um testemunho eloquente do seu talento ímpar como pintor ( Figura 1 ). A obra retrata o desenvolvimento da cardiologia no Brasil. Trata-se de um conjunto de três painéis de tinta acrílica sobre tela justapostos, medindo 6,30m de largura e 1,80m de altura, com personagens reais e imaginários que integram a narrativa de mais de um século da prática da cardiologia na maior nação dos trópicos. O mural de Flávio Tavares será um legado para a história da cardiologia brasileira assim como tem sido o mais famoso mural da história da cardiologia mundial pintado por Diego Rivera, em 1944, para o Instituto de Cardiologia na cidade do México.^1^

A medicina, presente dos deuses aos médicos para alívio do sofrimento dos homens, é representada como estatuário de forma monumental em plano avançado por Esculápio (Asclépio) e sua filha Higeia (Salus), que abrem espaço para os pioneiros da medicina social brasileira. O enredo tem como cenário a paisagem amazônica que, em suave e harmônica transição, é substituída pelo Castelo Mourisco da Fiocruz e pela ambiência do Brasil rural, subdesenvolvido, com casebres de taipa e, em sequência, pelo edifício do Instituto do Coração do Hospital das Clínicas da Universidade de São Paulo (InCor).

Com sua exuberante biodiversidade, a Amazônia simboliza a imensa fronteira para a pesquisa de possíveis princípios ativos empregados em medicina. As propriedades medicinais das plantas têm sido descritas por milênios, sendo um exemplo a dedaleira ( *Digitalis purpurea* ), planta empregada para tratar os males do coração. William Whittering, médico britânico descobridor das propriedades medicinais da *digitalis* presente na dedaleira, mereceu destaque na pintura. O gênio impressionista Vincent Van Gogh, igualmente, imortalizou a dedaleira em “O retrato do Dr. Gachet” *(Musée D’Orsay* – Paris), plasmando sua relação com a medicina e a arte. A magia da pintura permite confirmar a visão dos descobridores “nessa terra tudo que se planta dá”, e um frondoso salgueiro, base da síntese do ácido acetilsalicílico por Felix Hoffman, foi integrado à paisagem amazônica.

A biodiversidade e a sua utilidade para a ciência são uma temática inovadora, mas sempre presente na medicina, como se pode ver no dizer de Paracelso: “A medicina se fundamenta na natureza, a natureza é a medicina e somente naquela devem os homens buscá-la. A natureza é o mestre do médico, já que ela é mais antiga do que ele e ela existe dentro e fora do homem.” Tal fato se confirma na pesquisa de Sergio Henrique Ferreira, que liderou a síntese do peptídeo ativo presente no veneno da jararaca do mato. Esse veneno é a base para a produção do captopril, fármaco que mudou a história natural de enfermidades como hipertensão arterial e insuficiência cardíaca.

Os pesquisadores William Harvey, Daniel Hale Williams e Willem Einthoven representam os primórdios da cardiologia como ciência, para além dos símbolos gregos clássicos. *Sir* William Harvey, considerado o pai da cardiologia, desvendou os mistérios da circulação sanguínea, desafiando todos os conceitos da época, inclusive as crenças religiosas. A cardiologia mundial celebrará 400 anos da publicação do livro *De Motu Cordis* , o mais antigo paradigma científico da medicina, escrito por Harvey.^2^ Em 1893, o vanguardista cirurgião afro-americano Daniel Hale Williams realizou a primeira cirurgia bem-sucedida de peito aberto para reparar uma ferida no pericárdio.

Willem Einthoven, agraciado com o Prêmio Nobel de medicina em 1924, tem protagonismo ímpar na cardiologia e sua presença é obrigatória ao se contar a história da especialidade. Rubens Maciel destaca que Einthoven, ao receber o Prêmio Nobel de medicina a 8 de dezembro de 1925 em memorável conferência sobre o galvanômetro de corda e a medida da corrente de ação do coração, salientou: “um novo capítulo se abria no aprendizado das doenças do coração, não por obra de um só homem, mas pelo trabalho conjugado de muitos homens de talento que, espalhados pelo mundo e sem respeitar fronteiras políticas, convergiam seus esforços para um propósito comum: aumentar nosso conhecimento da doença, para alívio da humanidade sofredora”.^3^

Nos primórdios da cardiologia brasileira tivemos a influência do doutor piauiense Pedro Francisco da Costa Alvarenga que, no final do século XIX, escreveu sobre cardiopatia congênita e tornou-se uma celebridade da cardiologia francesa, tendo posteriormente retornado ao Brasil.^4^ A medicina praticada no século XIX até meados do século XX está bem representada no mural. À época, pouco podia ser feito para alterar o curso natural das doenças, restando tão somente o humanismo e a fé como alento para os enfermos.

A interação médico-paciente, imprescindível para o exercício da medicina, tem destaque no mural. Percebem-se as premissas semióticas do exame do aparelho cardiovascular – aferição do pulso e da pressão arterial e ausculta cardíaca. O francês René Leannec, ao inventar o estetoscópio, fez o cardiologista se diferenciar dos demais médicos por interpretar a “voz do coração”. A capacidade de distinguir os sons das bulhas, cliques, estalidos e sopros, correlacionando-os com as doenças do coração, passou a ser um dom almejado e um requisito para se compreender a verdadeira sinfonia executada pelo órgão da vida. A ausculta cardíaca é retratada de maneira original no mural, inspirado no movimento antropofágico de Oswald de Andrade: vê-se o cardiologista auscultando uma índia em uma oca. O simbolismo autóctone dessa imagem pictórica reafirma a necessidade da convivência harmônica dos povos de diversas culturas com o meio ambiente.

A vocação das mulheres na arte de cuidar é reconhecida desde o início desses séculos. Nos primórdios da cardiologia, contudo, a presença delas era muito restrita. Por isso, o esforço pioneiro das médicas Rita Lobato Velho Lopes e Anna Turan Machado Falcão impõe-se como forma de registrar a incomparável habilidade das mulheres para o exercício da medicina. A gaúcha Rita Lobato foi a primeira médica brasileira. Ingressou na Faculdade de Medicina do Rio de Janeiro, tendo concluído o curso na Faculdade de Medicina da Bahia. A outra pioneira, a médica paraense Anna Turan Machado Falcão, também teve sua notável trajetória de vida destacada. Formou-se em 19 de abril de 1887 em Nova York em uma turma composta por dez mulheres. Recebeu medalha de honra ao mérito, de ouro, aposta ao diploma. Apesar de não terem se dedicado à cardiologia, figuram no mural por serem inspiração para as médicas brasileiras.

Carlos Chagas, considerado por muitos o primeiro cardiologista moderno e pioneiro da ciência translacional por sua liderança na saúde pública brasileira na fase final da pandemia em 1919, é o personagem central do mural.^5^ Na pintura, o pesquisador e seus mentores Oswaldo Cruz e Miguel Couto dialogam com os pioneiros da cardiologia brasileira. O esforço na descrição da tripanossomíase americana, ou doença de Chagas, e a identificação cuidadosa do ambiente onde se difunde a endemia são retratados com riqueza de detalhes. Chagas e Oswaldo Cruz simbolizam a premência de investimento na medicina social, voltada para a atenção primária e a promoção da saúde. O estudo da cardiopatia chagásica deu destaque internacional à cardiologia brasileira e Anis Rassi representa esse modelo de liderança médica que muito contribuiu para o conhecimento dos fatores prognósticos dessa ainda importante endemia latino-americana.^6^

O início da década de 40, ambiente de forte ebulição cultural e social, marcou a fundação da SBC. No mural, emergem as figuras dos fundadores da SBC, Dante Pazzanese (primeiro presidente), Jairo Ramos e Genival Londres, que simbolizam uma era de ouro da medicina cardiovascular. O doutor Dante foi o aglutinador de um grupo extraordinário de médicos de várias regiões do país que já se destacavam no estudo das doenças do coração, sendo a criação da SBC uma consequência natural dessas ações. À época, Dante Pazzanese e Genival Londres já haviam fundado os Institutos Estaduais de Cardiologia de São Paulo e do Rio de Janeiro, respectivamente. Genival Londres publicou o primeiro livro sobre hipertensão arterial no Brasil e, à guisa de ilustração, registrou não existir tratamento eficaz para o mal, como destacado por Rafael Leite Luna.^7^

No mural, Jairo Ramos é o símbolo do compromisso perene com o avanço da pesquisa no Brasil e sua publicação. Foi o primeiro editor dos *Arquivos Brasileiros de Cardiologia* (ABC) e presidente da SBC. Hoje, os ABC são a publicação da cardiologia com maior fator de impacto em língua portuguesa, constituindo o registro vivo da evolução da cardiologia brasileira e o desaguadouro da produção científica nacional.^8^

A cardiologia da primeira metade do século XX foi inicialmente construída através de argúcia clínica apoiada pelo eletrocardiograma, pela radiografia do tórax e pela fonomecanocardiografia. Naquela época, os mestres franceses, ingleses e posteriormente os da escola mexicana de cardiologia foram as grandes referências para a formação dos nossos cardiologistas. O genial professor Ignácio Chaves, ao fundar o Instituto de Cardiologia do México, criou um ambiente único de ensino-pesquisa e assistência, que formou e influenciou gerações de brasileiros.

As Santas Casas foram o local de cuidar dos mais pobres com doença cardíaca. Pouco a pouco, porém, transformaram-se em centros de investigação cardiovascular e de ensino. O professor Nelson Botelho Reis introduziu na 6ª Enfermaria da Santa Casa do Rio de Janeiro o raciocínio clínico-hemodinâmico à beira do leito. Na 29ª Enfermaria da Santa Casa de Porto Alegre, o grande educador e líder Rubens Maciel promoveu uma nova visão da assistência e criou a pós-graduação médica.

A cardiologia de meados do século XX era ainda muito marcada pela doença reumática, que acometia crianças e adultos jovens e estava associada às condições sanitárias precárias dos brasileiros. O livro de Luiz Venere Décourt sobre doença reumática, lançado em 1965, representa um dos marcos das publicações médicas do nosso país. Luiz Venere Décourt foi um ícone da cardiologia, síntese da ciência e do humanismo necessários ao exercício da medicina. Abriu as portas para a moderna cardiologia brasileira. Sua trajetória ímpar foi imortalizada na criação do InCor, inaugurando uma nova perspectiva para a cardiologia nacional. Décourt e Zerbini foram os artífices da criação do InCor, um hospital público voltado à assistência, ao ensino e à pesquisa e que teve nas lideranças de Fulvio Pileggi e Adib Jatene a consolidação da inserção da cardiologia do Brasil, definitivamente, entre as mais respeitadas do mundo.

O notável avanço da cirurgia cardiovascular tem seu papel registrado, pois foi fundamental para o desenvolvimento da cardiologia brasileira. Euryclides de Jesus Zerbini e Adib Jatene, dois ícones e personalidades que transpuseram as fronteiras da cardiologia, foram os protagonistas. O doutor Zerbini, figura emblemática, para quem nada vencia o trabalho, realizou o primeiro transplante de coração da América Latina, logo após Christiaan Barnard, iniciando uma era de ouro na cirurgia cardíaca brasileira. O seu esforço foi recompensado. Hoje o Brasil tem o maior programa público de transplante de coração do mundo. Por sua vez, o doutor Jatene, para muitos o médico mais proeminente da sua geração, foi duas vezes ministro da saúde e é retratado no mural por sua vertente mais humana: devolver a alegria às mães de crianças que nascem com cardiopatia. O desafio de realizar a correção anatômica da transposição das grandes artérias foi superado pela perícia técnica do doutor Adib, um grande salto para a cirurgia cardiovascular infantil.

A cardiologia, ao aliar a arte da medicina à tecnologia, incorporou o progresso da ciência às necessidades da assistência, sendo o eletrocardiograma a expressão mais eloquente desse binômio. No Brasil, João Tranchesi, retratado no mural, simboliza a difusão da eletrocardiografia moderna. Com didática incomparável, democratizou o aprendizado do método. Como tributo, seus discípulos realizam anualmente no Congresso Brasileiro de Cardiologia a Sessão João Tranchesi, onde se discute o estado da arte na eletrocardiografia em sua homenagem. A contemporaneidade do método é indiscutível e seu emprego, na versão digital, constitui-se importante ferramenta da Telemedicina, ampliando o acesso em regiões remotas e provendo agilidade ao tratamento do infarto do miocárdio.

A mudança do perfil de adoecimento dos brasileiros, em parte decorrente da transição demográfica, com elevada incidência e prevalência da doença coronária, faz-se presente no mural. A mão espalmada no peito, tal qual descrito por Cossio-Levine,^9^ marca registrada do paciente anginoso, representa o sofrimento de centenas de milhares de brasileiros que morrem em decorrência dessa enfermidade. O cateterismo cardíaco, parte importante na estratégia diagnóstica e terapêutica desses enfermos, tem no doutor Eduardo Sousa, pioneiro da cinecoronariografia e da cardiologia intervencionista no Brasil, seu mais destacado precursor. Eduardo Sousa e Adib Jatene, parceiros de uma vida, foram os responsáveis pela consolidação do Instituto Dante Pazzanese de Cardiologia (IDPC) como importante centro de pesquisa, ensino e assistência no Brasil. No IDPC, foram realizadas as primeiras pesquisas com os *stents* farmacológicos em seres humanos no mundo. Impossível abordar a evolução da terapia da doença arterial coronária sem reverenciar René Favaloro, responsável pelo grande impulso da cirurgia de revascularização do miocárdio. Essas são contribuições originais de médicos do nosso hemisfério.

No alto do mural, em sua parte contemporânea, emerge a figura emblemática do professor Eugene Braunwald da Universidade de Harvard, uma lenda viva da cardiologia e talvez o nome de maior expressão da cardiologia moderna. Mesmo nonagenário, por seu saber e sua experiência, continua a influenciar a especialidade no mundo. Esse registro simboliza a necessidade do intercâmbio científico permanente com os centros mundiais para o desenvolvimento da cardiologia no Brasil.^10^ Abaixo, na parte inferior do painel, diversos nomes emblemáticos da cardiologia nacional são retratados, todos atentos a um enfermo que padece no leito. A cena se assemelha à “A lição de anatomia do Dr. Tulp” do lendário Rembrandt. Estão também retratados os cardiologistas Adriano Pondé, Rafael Leite Luna, Rachel Snitcovsky, Betina Ferro, Edgard Magalhães Gomes, Fulvio Pileggi, José Krieger, Ivo Nersralla, Siguemituzo Arie, Arnaldo Elian e Ênio Cantarelli. Vários desses ilustres médicos presidiram a SBC e todos foram determinantes para o desenvolvimento da cardiologia no Brasil.

Se olharmos para a disposição dos personagens, é possível perceber que eles estão distribuídos em uma linha do tempo ilusória, onde os responsáveis pelas primeiras descobertas estão à esquerda e os mais recentes, no extremo oposto à direita. A proporção dos personagens e seus tamanhos não guardam relação com sua hipotética importância histórica, mas integram um conjunto que deve dialogar com a ambiência da perspectiva artística. Segundo Flávio Tavares, é impossível registrar em uma pintura todas as personalidades que construíram a cardiologia brasileira, tendo sido um desafio instigante todo o esforço dedicado à construção de um enredo capaz de exprimir essa história.

A expressão figurativa artística tem seus primeiros registros na arte rupestre, sendo, talvez, sua primeira manifestação na humanidade. A necessidade humana de registrar o saber médico é visceral, chegando, em alguns casos, a pôr em risco a própria vida, como exemplos dos anatomistas Andreas Vesalius e Leonardo Da Vinci, que profanaram cadáveres impulsionados por uma curiosidade proibitiva para a época. Portanto, o casamento entre arte e medicina foi responsável pelo avanço e disseminação do saber médico. O Coração dos Trópicos eterniza sinteticamente em poucos metros e de forma alegórica a nossa cardiologia. Esse painel requereu um apurado trabalho de pesquisa e o resultado passou por diversos ajustes para harmonizar a história da cardiologia brasileira dentro de uma perspectiva artística capaz de compatibilizar os personagens reais com figurantes e enfermos, permitindo um mergulho em mais de um século do desenvolvimento da especialidade em nosso meio. Um universo de cores, luzes e sombras se combina para integrar os personagens ao cenário onírico da pintura. No dizer de Emilia Viotti da Costa, um povo sem história é um povo sem memória.
