# Supplementary material for: Pintando a História da Cardiologia do Brasil
Source: Arq Bras Cardiol. 2020 Dec 1;115(6):1047–50. [Article in Portuguese] doi: 10.36660/abc.20201133 (PMC8133712; doi:10.36660/abc.20201133)
Supplement: Translation

# Translation: Painting the History of Brazilian Cardiology

**Authors:** Marcelo Antônio Cartaxo Queiroga Lopes, Gláucia Maria Moraes de Oliveira, Evandro Tinoco Mesquita, Marcelo Dantas Tavares de Melo, Fernando Bacal

**Affiliations:** 1 Brazil Hospital Alberto Urquiza Wanderley, Cabedelo, PB - Brazil; 2 Brazil Universidade Federal do Rio de Janeiro, Rio de Janeiro, RJ – Brazil; 3 Brazil Universidade Federal Fluminense, Niterói, RJ - Brazil; 4 Brazil Universidade Federal da Paraíba, João Pessoa, PB - Brazil; 5 Brazil Universidade de São Paulo Faculdade de Medicina Hospital das Clínicas Instituto do Coração, São Paulo, SP – Brazil

**Keywords:** Cardiology/history, Cardiology/trends, Cardiologists, Medicine in the Arts/history, Paintings

The mural painted by the visual artist Flávio Tavares for the Brazilian Society of Cardiology (SBC), The Heart of the Tropics, is an eloquent witness of his unique talent as a painter ( Figure 1 ). The mural portrays the development of Brazilian cardiology. It comprises three juxtaposed acrylic-on-canvas panels, measuring 6.30m of length and 1.80m of height, with real and imaginary characters illustrating more than one century of the practice of cardiology in the largest nation of the tropics. Similarly to the most famous mural on the history of world cardiology painted by Diego Rivera in 1944 for the *Instituto de Cardiología de México* , the mural by Flávio Tavares is a legacy for the history of Brazilian cardiology.^1^

**Figure 1:** – Painting the History of Brazilian Cardiology.

Medicine, a gift from the Gods to physicians to relieve human suffering, is monumentally represented by figures, among which Aesculapius (Asclepius) and his daughter Hygieia (Salus) make way for the pioneers of Brazilian social medicine. In the background, in a smooth and harmonious transition, the Amazonian landscape is replaced by the *Mourisco* Castle from Fiocruz, a Brazilian underdeveloped rural setting, and then the building of the *Instituto do Coração* of the Hospital das Clínicas of the University of São Paulo (InCor).

With its exuberant biodiversity, the Amazonia symbolizes a huge frontier for the research of possible active principles used in medicine. The medicinal properties of plants have been described for millennia, an example being the foxglove plant ( *Digitalis purpurea* ), used to treat heart conditions. The British physician William Whittering, who has discovered the medicinal properties of *digitalis* present in the foxglove plant, stands out in the painting. Similarly, Vincent Van Gogh, the Impressionist genius, has immortalized foxglove in “The Portrait of Dr. Gachet” painting *(Musée D’Orsay* – Paris), shaping his relationship with medicine and art. The magic of the painting confirms the vision of the explorers that ours is a fertile land, and a leafy willow tree, the base for the acetylsalicylic acid synthesis by Felix Hoffman, was integrated into the Amazonian landscape.

Biodiversity and its use for science are an innovative theme, although it has always been present in medicine. According to Paracelsus, medicine is based on nature, and nature is medicine, which should only be pursued by men in nature; nature is the master of the physician, because nature is older than the physician and exists inside and outside men. This is supported by the research by Sergio Henrique Ferreira, who led the synthesis of the bioactive peptide found in the Bothrops jararaca venom. This venom is the base to produce captopril, a drug that has changed the natural history of illnesses, such as arterial hypertension and heart failure.

The researchers William Harvey, Daniel Hale Williams and Willem Einthoven represent the dawn of cardiology as a science, beyond the classical Greek symbols. Sir William Harvey, considered the father of cardiology, has unveiled the mysteries of blood circulation, challenging all the concepts of his time, including religious beliefs. Harvey has written the book *De Motu Cordis* , the oldest scientific paradigm of medicine, whose 400 years of publication is celebrated by world cardiology.^2^In 1893, Daniel Hale Williams, an avant-garde Afro-American surgeon, performed the first successful open-chest surgery to repair a pericardial wound.

Willem Einthoven, bestowed with the Nobel Prize of Medicine 1924, has played a unique role in cardiology, having mandatory presence in the history of that specialty. Rubens Maciel states that Einthoven, when receiving the Nobel Prize of Medicine on December, 8^th^, 1925, in a memorable conference about the string galvanometer and measurement of the cardiac action potential, has highlighted: “A new chapter has been opened in the study of heart diseases, not by the work of a single investigator, but by that of many talented men, who have not been influenced in their work by political boundaries and, distributed over the whole surface of the Earth, have devoted their powers to an ideal purpose, the advance of knowledge by which, finally, mankind suffering is helped”.^3^

The beginning of Brazilian cardiology has been influenced by the physician Pedro Francisco da Costa Alvarenga, from the Brazilian state of Piauí, who, at the end of the 19^th^century, wrote about congenital heart disease and became a celebrity of French cardiology, returning later to Brazil.^4^Medicine practiced from the 19^th^century to the mid-20^th^century is well portrayed in the mural. At that time, little could be done to change the natural course of diseases, leaving only humanism and faith as a relief for the sick.

The patient-doctor interaction, essential for the practice of medicine, is highlighted in the mural. The semiotic assumptions of the cardiovascular system examination – pulse and blood pressure measurement and cardiac auscultation – are represented. The Frenchman René Leannec, by inventing the stethoscope, enabled cardiologists to differentiate from other physicians by interpreting the “voice of the heart”. The ability to distinguish heart sounds, clicks, and murmurs, and to correlate them with heart diseases became a desired gift and a requirement for understanding the true symphony of the heart. Cardiac auscultation is originally portrayed in the mural, inspired by the Oswald de Andrade’s anthropophagic movement: a cardiologist is portrayed auscultating a South American Indian in her native household. The aboriginal symbolism of the image reassures the need for the harmonious coexistence of people from different cultures with the environment.

The vocation of women for caring has long been recognized. However, in the beginning, their presence in cardiology was limited. Thus, the pioneering effort of the female physicians Rita Lobato Velho Lopes and Anna Turan Machado Falcão is a way to register the incomparable ability of women for the practice of medicine. Rita Lobato, from the Brazilian state of Rio Grande do Sul, was the first Brazilian female physician. She started medical school in Rio de Janeiro and concluded it in Bahia. The other pioneer, the female physician from the state of Pará, Anna Turan Machado Falcão, also had her remarkable life trajectory portrayed. She graduated in April 1887 in New York with only nine other women in her class. When receiving her diploma, she was awarded the golden medal of Honor to Merit. Although they were not cardiologists, they are portrayed in the mural because they are an inspiration to all Brazilian female physicians.

Carlos Chagas, considered the first modern cardiologist and the translational science pioneer because of his leadership in Brazilian public health in the 1919 pandemic, is the central character in the mural.^5^In the painting, he and his mentors Oswaldo Cruz and Miguel Couto are portrayed having a conversation with Brazilian cardiology pioneers. The endeavor to describe American trypanosomiasis, or Chagas disease, as well as the thorough identification of the environment where the endemic spreads are portrayed in detail. Chagas and Oswaldo Cruz symbolize the urgency of investing in social medicine, directed at primary attention and health promotion. The study of Chagas heart disease brought international attention to Brazilian cardiology, and Anis Rassi represents a model of medical leadership that has contributed to the knowledge of the prognostic factors of that important Latin American endemic.^6^

In the beginning of the 1940s, a period of strong cultural and social effervescence, the SBC was founded. In the mural, the figures of Dante Pazzanese (the first president of the SBC), Jairo Ramos and Genival Londres, founders of the SBC and symbols of a golden era of cardiovascular medicine, emerge. Dante Pazzanese was the catalyst of an extraordinary group of physicians from several Brazilian regions who already stood out in the study of heart diseases, and the creation of the SBC was a natural consequence of those actions. By that time, Dante Pazzanese and Genival Londres had already founded the São Paulo and Rio de Janeiro State Institutes of Cardiology, respectively. Genival Londres published the first book about arterial hypertension in Brazil and, by way of illustration, he reported there was no effective treatment for the condition, as highlighted by Rafael Leite Luna.^7^

In the mural, Jairo Ramos is the symbol of perennial commitment to the advance and publication of research in Brazil. He was the first editor of the cardiology journal *Arquivos Brasileiros de Cardiologia* (ABC) and president of the SBC. Currently, the ABC is the journal with the highest impact factor in the Portuguese language, constituting the live registry of the evolution of Brazilian cardiology and the outreach channel for the Brazilian scientific production.^8^

The cardiology of the first half of the 20^th^century was built on clinical acuity supported by electrocardiography, chest radiography and phonomechanocardiography. At that time, the French, English, and later Mexican masters were the great references for the formation of our cardiologists. The brilliant professor Ignácio Chaves, by founding the *Instituto de Cardiología de México* , has created a unique environment of teaching, researching and healthcare, forming and influencing generations of Brazilians.

The Santa Casa philanthropic hospitals were initially the place to treat poor people with heart disease, but little by little they were turned into cardiovascular research and teaching centers. Professor Nelson Botelho Reis has introduced the clinical-hemodynamic reasoning at bedside on the 6^th^Ward of the Santa Casa from Rio de Janeiro. On the 29^th^Ward of the Santa Casa from Porto Alegre, the great educator and leader Rubens Maciel has promoted a new vision of healthcare and created the medical postgraduation program.

The cardiology of the mid-20^th^century was still very impacted by rheumatic disease affecting children and young adults, which was associated with the then precarious sanitary conditions of Brazil. The book by Luiz Venere Décourt about rheumatic disease, issued in 1965, represents a landmark of the medical publications in our country. Luiz Venere Décourt has been an icon of cardiology, synthesizing the science and humanism necessary to the practice of medicine. He opened the doors for Brazilian modern cardiology. His unique trajectory has been immortalized with the creation of InCor, which inaugurated a new perspective for Brazilian cardiology. Décourt and Zerbini were the authors of InCor, a public hospital directed at healthcare provision, teaching and research, which, under the leadership of Fulvio Pileggi and Adib Jatene, has definitely consolidated the insertion of Brazilian cardiology among the most respected ones worldwide.

Since cardiovascular surgery has been essential for the development of Brazilian cardiology, its remarkable advance is portrayed in the mural. Euryclides de Jesus Zerbini and Adib Jatene, two icons and personalities who have crossed the frontiers of cardiology, were the leading figures. Zerbini, an emblematic person who praised hard-working, performed the first heart transplantation in Latin America, right after Christiaan Barnard, inaugurating a golden era in Brazilian cardiac surgery. His effort payed off. Currently Brazil has the largest public program of heart transplantation in the world. Adib Jatene, considered the most prominent physician of his generation, has been Brazil Minister of Health twice and is portrayed in the mural according to his most human quality: returning joy to mothers of children with congenital heart diseases. The challenge of performing the anatomical correction of the transposition of the great arteries has been overcome by Jatene’s technical skill, a great leap for pediatric cardiovascular surgery.

Cardiology, by uniting the art of medicine and technology, has incorporated the progress of science to the needs of healthcare provision, the electrocardiogram being the most eloquent expression of that binomial. João Tranchesi, another character portrayed in the mural, symbolizes the diffusion of modern electrocardiography in Brazil. With incomparable didacticism, he has popularized the learning of that technique. As a tribute, his disciples hold annually the João Tranchesi Session at the Brazilian Congress of Cardiology, where state-of-the-art electrocardiography is discussed in his honor. The contemporaneity of the technique is indisputable, and its digital version is an important Telemedicine tool, reaching remote regions and speeding up the treatment of myocardial infarction.

The change in the illness profile of Brazilians, partially due to demographic transition, with high incidence and prevalence of coronary disease, is represented in the mural. The clenched fist held over the chest, the Levine’s sign,^9^a registered mark of the patient with angina, represents the suffering of hundreds of thousands of Brazilians who die from that condition. Cardiac catheterization, which plays an important role in the diagnostic and therapeutic strategy of those patients, has Eduardo Sousa, a pioneer of coronary cineangiography and interventional cardiology in Brazil, as its most remarkable precursor. Eduardo Sousa and Adib Jatene, lifelong partners, have been responsible for the consolidation of the Instituto Dante Pazzanese de Cardiologia (IDPC) as an important research, teaching and healthcare center in Brazil. The first studies in the world on the use of drug-eluting stents in human beings have been conducted at the IDPC. When addressing the evolution of the therapy for coronary artery disease, René Favaloro, responsible for the great boost on coronary artery bypass grafting, should be revered. Those are the contributions of physicians from the southern hemisphere.

At the, the emblematic figure of Professor Eugene Braunwald from Harvard University, a living legend of cardiology and perhaps the most expressive name of modern cardiology, emerges. Even nonagenarian, he continues to influence the specialty worldwide because of his knowledge and experience. His presence in the mural symbolizes the need for permanent scientific exchange with other centers around the world for the development of cardiology in Brazil.^10^In the bottom of the mural, several emblematic figures of Brazilian cardiology are portrayed, all of them taking care of bedridden patients. The scene reminds us of Rembrandt’s “The Anatomy Lesson of Dr. Tulp”. In addition, the cardiologists Adriano Pondé, Rafael Leite Luna, Rachel Snitcovsky, Betina Ferro, Edgard Magalhães Gomes, Fulvio Pileggi, José Krieger, Ivo Nersralla, Siguemituzo Arie, Arnaldo Elian and Ênio Cantarelli are portrayed. All these remarkable physicians have had a significant role in the development of cardiology in Brazil and some have even been presidents of the SBC.

The characters are displayed according to a fictional timeline, in which those responsible for the first discoveries are on the left-hand side and the most recent ones, on the right-hand side. The proportion of the characters and their sizes have no relation to their hypothetical historical importance but comprise a set that interacts with artistic perspective. According to the painter Flávio Tavares, it is not possible to portray in a painting all the personalities of Brazilian cardiology, and much effort was put into the construction of a plot capable of expressing that history, a stimulating challenge.

Artistic figurative expression has been first registered in rupestrian art, which might have been the first artistic manifestation of mankind. The human need for registering medical knowledge is visceral, and some individuals have even put their own lives at risk for that, such as anatomists Andreas Vesalius and Leonardo Da Vinci, who have desecrated corpse driven by a curiosity prohibited at their times. Thus, the combination of art and medicine has accounted for the advance and spread of medical knowledge. The mural ‘Heart of the Tropics’ perpetuates in a few meters and in an allegorical way our cardiology. This mural required careful research, whose result underwent several adjustments to harmonize the history of Brazilian cardiology from an artistic perspective capable of mixing real characters with imaginary and sick people, providing a dive into over a century of the development of Brazilian cardiology. A universe of colors, lights and shade has been combined to incorporate the characters into the dreamlike scenario of painting. According to Emilia Viotti da Costa, a nation without history is a nation without memory.
